# Analysis of prognostic factors in critically ill patients with COVID-19

**DOI:** 10.1371/journal.pone.0302248

**Published:** 2024-06-27

**Authors:** Klaudia Bartoszewicz, Mateusz Bartoszewicz, Wojciech Gradkowski, Samuel Stróż, Anna Stasiak-Barmuta, Sławomir Lech Czaban

**Affiliations:** 1 Department of Clinical Immunology, Medical University of Bialystok, Bialystok, Poland; 2 Department of Anaesthesiology and Intensive Care, Medical University of Bialystok, Bialystok, Poland; Children’s National Hospital, George Washington University, UNITED STATES

## Abstract

The Coronavirus Disease 2019 (COVID-19) has caused a global health crisis. Mortality predictors in critically ill patients remain under investigation. A retrospective cohort study included 201 patients admitted to the intensive care unit (ICU) due to COVID-19. Data on demographic characteristics, laboratory findings, and mortality were collected. Logistic regression analysis was conducted with various independent variables, including demographic characteristics, clinical factors, and treatment methods. The study aimed to identify key risk factors associated with mortality in an ICU. In an investigation of 201 patients comprising non-survivors (n = 80, 40%) and Survivors (n = 121, 60%), we identified several markers significantly associated with ICU mortality. Lower Interleukin 6 and White Blood Cells levels at both 24- and 48-hours post-ICU admission emerged as significant indicators of survival. The study employed logistic regression analysis to evaluate risk factors for in-ICU mortality. Analysis results revealed that demographic and clinical factors, including gender, age, and comorbidities, were not significant predictors of in-ICU mortality. Ventilator-associated pneumonia was significantly higher in Survivors, and the use of antibiotics showed a significant association with increased mortality risk in the multivariate model (OR: 11.2, p = 0.031). Our study underscores the significance of monitoring Il-6 and WBC levels within 48 hours of ICU admission, potentially influencing COVID-19 patient outcomes. These insights may reshape therapeutic strategies and ICU protocols for critically ill patients.

## Introduction

The Coronavirus Disease 2019 (COVID-19), caused by the SARS-CoV-2 virus, emerged in late 2019 and rapidly evolved into a global pandemic, impacting healthcare systems and economies worldwide [1]. While most infected patients experience mild to moderate symptoms, a substantial fraction develop severe disease, demanding ICU admission and leading to significant mortality rates [2]. One of the most alarming aspects of the COVID-19 pandemic has been its impact on mortality, particularly among certain high-risk groups [3]. According to the World Health Organization (WHO), millions of confirmed cases and deaths have been attributed to COVID-19 worldwide [4]. Mortality rates have varied by region, age group, and presence of underlying conditions, among other factors [5].

Several studies have been conducted to identify risk factors associated with COVID-19 mortality [3, 6]. Age, for instance, has been consistently identified as a strong predictor, with older individuals facing higher risks of severe outcomes [5]. Other factors like comorbidities, including diabetes, hypertension, and cardiovascular diseases, have also been shown to increase the risk of mortality [7]. Some studies have additionally focused on biomarkers, such as elevated D-dimer levels, C-reactive protein, and decreased lymphocyte counts as potential indicators of severe outcomes [8–10]. However, much of this research is generalized and does not specifically delve into predictors of mortality in critically ill patients, a gap this study aims to fill.

Severe manifestations of COVID-19 are typified by cytokine storm syndrome [11]. The cytokine storm is a critical and potentially lethal systemic inflammatory response. This phenomenon is characterized by the excessive and uncontrolled release of pro-inflammatory cytokines and chemokines by immune effector cells. In the context of COVID-19, the cytokine storm has gained particular attention due to its role in developing disease severity and complications [12]. Clinically, the cytokine storm could manifest as severe respiratory distress due to the development of ARDS, multi-organ failure, and coagulopathy, among other complications [12]. It is a major cause of morbidity and mortality in severe cases of COVID-19 and requires early recognition and treatment.

ARDS is a life-threatening form of respiratory failure. It is marked by the rapid onset of widespread inflammation in the lungs, alveolar damage, and severe hypoxemia. The pathophysiology of ARDS in COVID-19 is complex and multifaceted, involving direct viral-induced lung injury, dysregulated immune response, and endothelial damage [12].

Among the pro-inflammatory cytokines, IL-6 is commonly considered the most important pro-inflammatory cytokine in terms of its association with the pathogenesis of severe COVID-19 [11, 12]. Elevated levels of IL-6 have been consistently associated with disease severity and adverse outcomes [13].

Identifying prognostic factors is important to improve outcomes. With the ability to predict which patients are at higher mortality risk, healthcare providers can implement early and aggressive intervention strategies. Moreover, understanding these predictors can inform public health policies and contribute to developing clinical guidelines, thereby improving the standard of care for COVID-19 patients. The study aims to determine the mortality risk factors in critically ill patients with COVID-19.

## Materials and methods

### Study design and population

We conducted a retrospective cohort study at a single medical centre, adhering to STROBE guidelines. From March 3, 2020, to July 1, 2021, 235 adult patients diagnosed with COVID-19 were treated in the Intensive Care Unit (ICU) at the University Clinical Hospital in Bialystok, Poland. Data were collected from the electronic medical records from September 1 to November 30, 2021. Other research results regarding this group of patients [14]. The research investigator retrieved all the patient’s data from the database in an Excel sheet and revised, cleaned, and coded the data. The study included participants at least 18 years old with a verified acute COVID-19 infection, confirmed through reverse transcription-polymerase chain reaction tests of nasal and throat swabs or lower respiratory secretions and ICU admission for SARS-CoV-2 infection.

**Fig 1:**
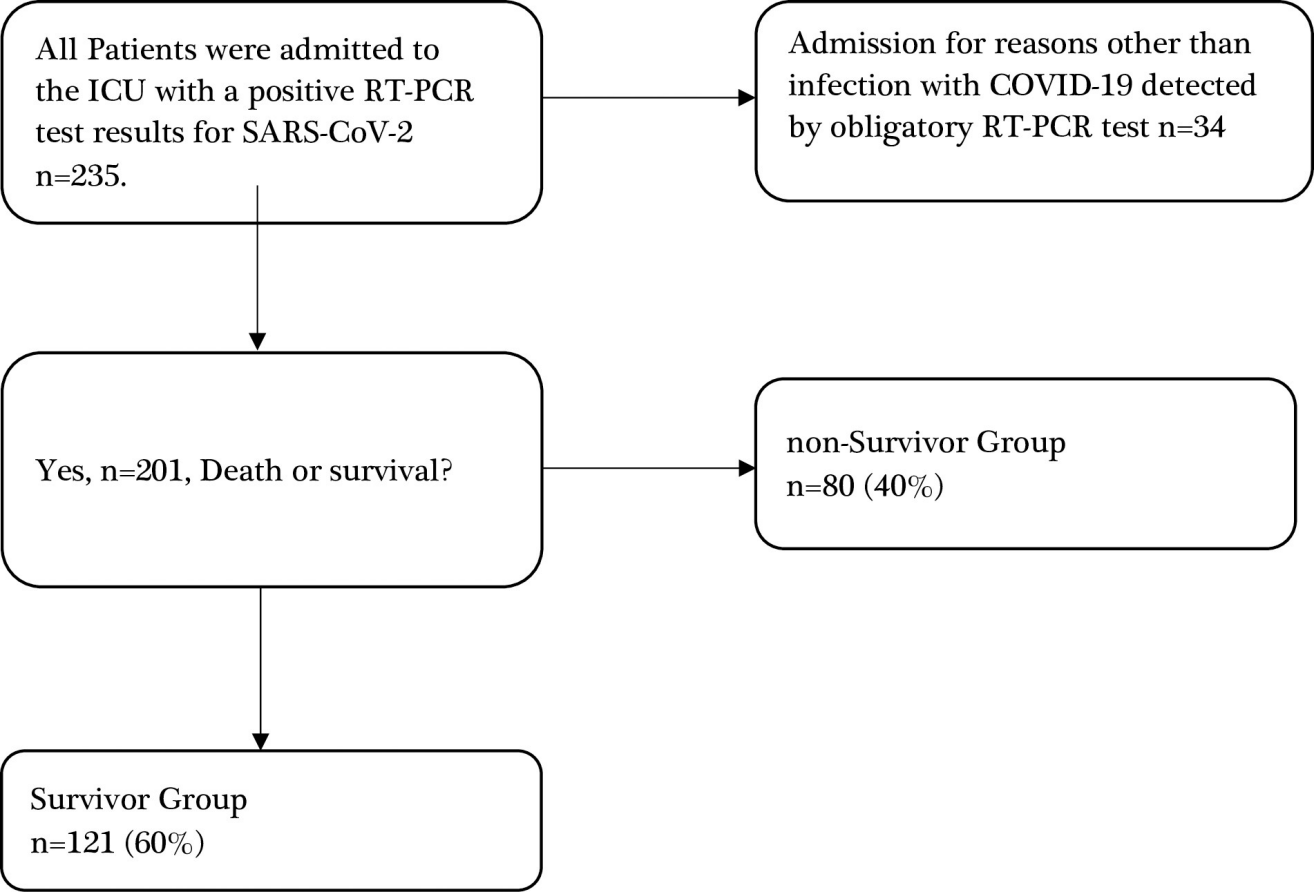
Flowchart of patient screening and inclusion. ICU: intensive care unit. SARS-CoV-2: severe acute respiratory syndrome coronavirus 2; COVID-19: coronavirus disease 2019; RT-PCR: reverse transcription-ploymerase chain reaction.

Excluded from this investigation were pregnant women and individuals in the ICU for non-COVID-19-related reasons, like elective surgeries or other emergency conditions. Consequently, the study comprised 201 participants, 80 (40%) of whom were categorized in the non-Survivor group. Details of the inclusion and exclusion criteria can be found in Fig 1’s flowchart.

A single diagnostic laboratory at the Medical University of Bialystok, Poland, was used to avoid technical variables affecting laboratory results.

During the study period, the diagnostic laboratory of the Medical University of Bialystok employed two specific diagnostic tests: the Allplex 2019nCoV Assay (Seegene Inc., Seoul, South Korea) and the COVID-19 Real Time Multiplex RT-PCR Kit (Labsystems Diagnostics Oy, Vantaa, Finland). Both tests were cross-validated in the laboratory to ensure consistency and reproducibility of results. More information about the analysis of RT-PCR test results for SARS-CoV-2 diagnostics done in this paper is available in this research [15]. PCT was determined using the immunoassay method. IL-6 concentration was determined using the ELISA method. CRP concentration in blood serum was analyzed using the turbidimetric method. Morphology was analyzed using the automatic analyzer.

### Statistical analysis

Data from the patients were entered into a pre-established institutional database. A t-test or a chi-square test was employed to evaluate the relationship between various factors, reporting the associated p-values. Logistic regression was also used to identify significant predictors affecting patient outcomes. A Logistic regression model included all variables in the univariate regression analysis with a p-value of less than 0.1. The aim was to determine factors independently predictive of illness progression. Two-tailed test, a p-value <0.05, was considered statistically significant. All statistical computations were conducted using R software version 4.1.1.

## Results

Table 1 presents the baseline and demographic characteristics of the 201 study participants, categorized into two groups: non-Survivors (n = 80, 40%) and Survivors (n = 121, 60%). No significant differences were observed between the Non-Survivors and Survivors in terms of average BMI (p = 0.474), sex distribution (p = 0.771), and mean age (p = 0.379). The prevalence of Diabetes Mellitus (DM), Atrial Fibrillation (AF), Hypertension (HT), and Chronic Heart Failure (CHF) were similar between the two groups (p>0.05 for all). Upon admission, APACHE II scores and Glasgow Coma Scale (GCS) were not significantly different between the two groups (p = 0.615 and p = 0.938, respectively). Inflammatory markers such as CRP, INR, and Interleukin 6 also showed no statistically significant differences (p>0.05). The length of stay (LOS) in ICU and the use of mechanical ventilation (MV) were similar in both groups (p = 0.551 and p = 0.450). Treatment modalities like the infusion of neuromuscular blocking agents (NMBAs) and corticosteroids did not vary significantly between the Non-Survivors and Survivors (p = 0.284 and p = 0.374, respectively). Ventilator-associated pneumonia (VAP) was significantly higher in the Survivor group (p = 0.048). Interleukin 6 levels after 48 hours of ICU hospitalization were significantly lower in the Survivor group (p<0.001). White Blood Cell counts after 24 and 48 hours of ICU hospitalization were significantly lower in the Survivor group (p = 0.012 and p = 0.022, respectively). Within 24 hours of ICU admission, non-survivors had significantly lower neutrophil percentages (60.3 ± 34.9 vs. 82.6 ± 19.4, p = 0.038), higher Interleukin-6 levels (448.4 ± 451.0 pg/mL vs. 206.1 ± 327.9 pg/mL, p = 0.050), and elevated white blood cell counts (15.9 ± 13.4 ×10^3^/μL vs. 11.4 ± 6.1 ×10^3^/μL, p = 0.012). These differences were sustained at 48 and 72 hours, most notably with Interleukin-6 levels being substantially higher in non-survivors at 48 hours (447.5 ± 390.8 pg/mL vs. 119.9 ± 154.5 pg/mL, p<0.001) and white blood cell counts consistently elevated across all time points (p = 0.022 at 48 hours, p = 0.034 at 72 hours).

**Table 1:** Characteristics of patients with COVID-19 at ICU admission, risk factors for death, disease course, treatment.

Headcount	non-Survivor n = 80 (40%)	Survivor n = 121 (60%)	All n = 201	*p*-value
**Baseline and demographic**				
Average BMI (± SD)	31.9 (7.4)	33.7 (21.1)	33.0 (17.0)	0.474
Female—no. (%)	36 (45.0)	51 (42.1)	87 (43.3)	0.771
Mean age (± SD)—years	67.0 (11.6)	65.5 (12.4)	66.1 (12.1)	0.379
DM	25 (31.2)	35 (28.9)	60 (29.9)	0.754
AF	11 (13.8)	17 (14.0)	28 (13.9)	1.000
HT	50 (62.5)	71 (59.2)	121 (60.5)	0.660
Obesity	17 (21.5)	26 (21.7)	43 (21.6)	1.000
CHF	18 (22.8)	28 (23.1)	46 (23.0)	1.000
**On arrival in the ICU**				
Mean APACHE II (± SD)	29 (8)	29 (8)	29 (8)	0.615
Mean GCS (+SD)	6.8 (5.4)	6.7 (5.3)	6.8 (5.3)	0.938
CRP (± SD) mg/L	81.1 (88.5)	85.5 (91.6)	83.7 (90.2)	0.735
INR (± SD)	1.3 (0.2)	1.4 (0.3)	1.4 (0.3)	0.739
Interleukin 6 (± SD) pg/mL	580.9 (1031.3)	372.5 (763.9)	451.4 (876.9)	0.186
White Blood Cells (×10^3^/μL) (±SD)	13.6 (11.5)	12.4 (8.0)	12.9 (9.5)	0.396
Neutrophils Percent (± SD)	69.2 (32.0)	79.2 (21.2)	75.2 (26.4)	0.071
Absolute neutrophils (×10^3^/μL)	11.2 (7.2)	10.1 (6.7)	10.5 (6.9)	0.467
Procalcitonin (± SD) ng/mL	2.5 (9.8)	2.1 (7.4)	2.3 (8.4)	0.780
**During hospitalization**				
Mean LOS at ICU (± SD)—days	13.4 (10.2)	12.5 (10.1)	12.8 (10.1)	0.551
MV—no. (%)	71 (88.8)	112 (92.6)	183 (91.0)	0.450
The average MV duration (± SD)—days	11.2 (8.8)	11.2 (9.1)	11.2 (9.0)	0.991
Infusion of NMBAs at least one day (%)	50 (62.5)	85 (70.2)	135 (67.2)	0.284
Corticosteroids—no. (%)	68 (85.0)	109 (90.1)	177 (88.1)	0.374
Antibiotics—no. (%)	69 (86.2)	113 (93.4)	182 (90.5)	0.138
Prone Position—no. (%)	33 (41.2)	46 (38.0)	79 (39.3)	0.661
Bacterial coinfecion—no. (%)	52 (65.0)	89 (73.6)	141 (70.1)	0.211
Fungal coinfecion—no. (%)	20 (25.0)	32 (26.4)	52 (25.9)	0.870
MDR—no. (%)	48 (60.0)	83 (68.6)	131 (65.2)	0.457
Sensitive—no. (%)	4 (5.0)	6 (5.0)	10 (5.0)	0.457
VAP—no. (%)	20 (25.0)	47 (38.8)	67 (33.3)	0.048
ARDS Mild	6 (7.5)	14 (11.6)	20 (10.0)	0.842
ARDS Moderate	31 (38.8)	45 (37.2)	76 (37.8)	
ARDS Not ARDS	4 (5.0)	5 (4.1)	9 (4.5)	
ARDS Severe	39 (48.8)	57 (47.1)	96 (47.8)	0.842
D-dimer (± SD)	3.9 (4.6)	3.9 (4.3)	3.9 (4.4)	0.985
BSI—no. (%)	15 (18.8)	28 (23.1)	43 (21.4)	0.487
Chronic organ insufficiency or immune compromise—no. (%)	52 (65.0)	76 (62.8)	128 (63.7)	0.767
**After 24 hours of ICU hospitalization**				
CRP (± SD) mg/L	59.0 (72.1)	76.2 (73.9)	69.3 (73.4)	0.177
D-dimer (± SD)	5.1 (5.7)	5.2 (5.1)	5.2 (5.3)	0.928
INR (± SD)	1.3 (0.2)	1.4 (0.3)	1.4 (0.3)	0.265
Interleukin 6 (± SD) pg/mL	448.4 (451.0)	206.1 (327.9)	297.0 (391.5)	0.050
Absolute neutrophils (×10^3^/μL)	10.9 (5.1)	10.4 (5.6)	10.6 (5.4)	0.827
Neutrophils Percent (± SD)	60.3 (34.9)	82.6 (19.4)	74.6 (27.5)	0.038
Procalcitonin (± SD) ng/mL	1.3 (2.9)	2.2 (7.3)	1.9 (6.0)	0.409
White Blood Cells (×10^3^/μL) (±SD)	15.9 (13.4)	11.4 (6.1)	13.2 (10.0)	0.012
**After 48 hours of ICU hospitalization**				
CRP (± SD) mg/L	47.8 (68.6)	55.7 (65.5)	52.6 (66.6)	0.499
D-dimer (± SD)	4.3 (5.5)	4.7 (4.6)	4.6 (4.9)	0.805
INR (± SD)	1.3 (0.2)	1.3 (0.3)	1.3 (0.2)	0.166
Interleukin 6 (± SD) pg/mL	447.5 (390.8)	119.9 (154.5)	218.1 (287.6)	<0.001
Absolute neutrophils (×10^3^/μL)	11.7 (6.1)	9.0 (5.8)	9.8 (5.9)	0.242
Neutrophils Percent (± SD)	64.9 (30.8)	78.7 (17.2)	74.5 (22.6)	0.109
Procalcitonin (± SD) ng/mL	0.9 (1.5)	1.7 (5.0)	1.3 (4.0)	0.272
White Blood Cells (×10^3^/μL) (±SD)	16.4 (14.1)	12.0 (6.9)	13.9 (10.8)	0.022
**After 72 hours of ICU hospitalization **				
CRP (± SD) mg/L	37.5 (46.3)	52.5 (54.6)	46.1 (51.6)	0.109
D-dimer (± SD)	3.8 (3.9)	4.7 (4.3)	4.3 (4.1)	0.474
INR (± SD)	1.3 (0.4)	1.3 (0.2)	1.3 (0.3)	0.770
Interleukin 6 (± SD) pg/mL	476.8 (1084.6)	736.6 (1233.5)	621.8 (1163.8)	0.474
Absolute neutrophils (×10^3^/μL)	12.7 (4.1)	12.6 (6.4)	12.6 (5.8)	0.961
Neutrophils Percent (± SD)	58.8 (37.1)	68.8 (27.8)	66.2 (30.0)	0.460
Procalcitonin (± SD) ng/mL	0.7 (1.2)	1.0 (2.1)	0.9 (1.8)	0.353
White Blood Cells (×10^3^/μL) (±SD)	17.7 (14.1)	13.3 (6.9)	15.2 (10.7)	0.034

The results are reported as a number (percentage) for categorical variables and median [IQR] and SD for continuous variables. DM: Diabetes Mellitus; AF: Atrial Fibrillation; HT: Hypertension; CHF: Chronic Heart Failure; APACHE II: Acute Physiology and Chronic Health Evaluation II; NMBAs: neuromuscular blocking agents; LOS: length of stay; ICU: Intensive Care Unit; VAP: ventilator-associated pneumonia, BSI: Bloodstream infection, 1 Median (IQR); n (%), 2 Wilcoxon rank sum test; Fisher’s exact test; Pearson’s Chi-squared test; Wilcoxon rank sum exact test, 3 False discovery rate correction for multiple testing

Table 2 presents the results from the logistic regression analysis examining risk factors associated with in-ICU mortality. Univariate Analysis: Among demographic parameters, none of the assessed variables, including Gender, Age, BMI, and underlying medical conditions such as DM, AF, and CHF, were significantly associated with in-ICU mortality.

**Table 2:** Logistic regression of factors associated with in-ICU mortality.

Independent variables	Univariate Analysis	Multivariate Analysis
	OR (95%CI), p-value	OR (95%CI), p-value
Female	1.12 (0.635–1.98, p = 0.690)	
Age	0.989 (0.966–1.01, p = 0.378)	
BMI	1.01 (0.984–1.03, p = 0.508)	
DM	0.895 (0.484–1.66, p = 0.725)	
AF	1.03 (0.453–2.32, p = 0.952)	
HT	0.869 (0.486–1.55, p = 0.637)	
OBESITY	1.01 (0.506–2.01, p = 0.980)	
CHF	1.02 (0.520–2.00, p = 0.953)	
APACHE II	0.991 (0.957–1.03, p = 0.613)	
White Blood Cells	0.987 (0.959–1.02, p = 0.397)	
Interleukin 6	1.000 (0.999–1.00, p = 0.194)	
CRP	1.00 (0.997–1.00, p = 0.734)	
Absolute neutrophils	0.978 (0.921–1.04, p = 0.464)	
Neutrophils Percent	1.014 (0.998–1.03, p = 0.078)	1.01 (0.998–1.03, p = 0.074)
Procalcitonin	0.995 (0.963–1.03, p = 0.779)	
LOS at ICU	0.992 (0.965–1.02, p = 0.550)	
MV	1.58 (0.598–4.16, p = 0.357)	
MV duration	1.00 (0.969–1.03, p = 0.991)	
Prone Position	0.874 (0.491–1.56, p = 0.646)	
Infusion of NMBAs at least one day	1.42 (0.780–2.57, p = 0.253)	
Corticosteroids	1.60 (0.681–3.77, p = 0.280)	
ANTIBIOTICS	2.252 (0.863–5.87, p = 0.097)	11.2 (1.2–101.66, p = 0.031)
VAP	1.91 (1.021–3.56, p = 0.043)	1.0825 (0.43–2.7, p = 0.87)
BSI	1.30 (0.646–2.63, p = 0.458)	
MDR	1.51 (0.814–2.81, p = 0.190)	
Bacterial coinfection	1.50 (0.812–2.76, p = 0.196)	
Fungal coinfection	1.08 (0.564–2.06, p = 0.819)	

APACHE II Acute Physiology and Chronic Health Evaluation II, ICU Intensive Care Unit, LOS length of stay, MV mechanical ventilation, BMI Body Mass Index, NMBA neuromuscular blocking agent, VAP Ventilator-Associated Pneumonia, BSI Bloodstream Infection, DM: Diabetes Mellitus, AF: Atrial Fibrillation, HT: Hypertension, CHF: Chronic Heart Failure, OR: Odds Ratio.

Only the neutrophil percentage was almost on the significance level in the univariate Analysis (OR: 1.014, 95% CI: 0.998–1.03, p = 0.078), similarly in the multivariate analysis (OR: 1.01, 95% CI: 0.998–1.03, p = 0.074). Ventilator-associated pneumonia (VAP) showed a significant association with in-ICU mortality (OR: 1.91, 95% CI: 1.021–3.56, p = 0.043), but this association did not remain significant in the multivariate analysis (OR: 1.0825, 95% CI: 0.43–2.7, p = 0.87). The use of antibiotics did not reach a level of statistical significance (OR: 2.252, 95% CI: 0.863–5.87, p = 0.097). However, in the multivariate analysis, the association became statistically significant (OR: 11.2, 95% CI: 1.2–101.66, p = 0.031).

## Discussion

Our study investigated various clinical and demographic variables to assess their potential impact on ICU mortality in 201 patients, non-Survivors (n = 80, 40%) and Survivors (n = 121, 60%). Traditional demographic and clinical parameters such as BMI, sex, age, and prevalent comorbidities showed no significant differences between the groups. We observed that VAP has a significant influence on in-ICU mortality. Interestingly, VAP occurrence was higher in Survivors. Initially, key inflammatory markers, including CRP and Il-6, did not differ significantly. However, over time, the most significant markers associated with survival were reduced levels of Il-6 after 24 and 48 hours and lower WBC counts after 24 and 48 hours of ICU admission.

The absence of significant differences in age, BMI, and pre-existing conditions indicate that these factors, usually considered crucial in determining outcomes, might be less predictive in ICU settings for COVID-19 patients, similar in research [6]. However, this is counter to other existing studies [16–18]. These results underscore the complexity of COVID-19’s impact.

Findings highlight the role of the patient’s immune response, particularly the inflammatory response, in influencing outcomes. The marked elevation of Il-6 in Non-Survivors is connected with cytokine storm, where an excessive immune response leads to tissue damage and organ failure. Furthermore, the higher WBC counts in Non-Survivors could indicate ongoing infection or inflammation, contributing to adverse outcomes. Lower Il-6 and WBC counts in survivors indicate a more controlled immune response, less likely to result in harmful inflammatory cascades [19]. It could be crucial for future targeted anti-inflammatory treatments for COVID-19 [11, 12, 20]. Medical teams could consider placing more emphasis on monitoring and controlling inflammatory markers within the first 48 hours of ICU admission. Il-6 and WBC can be used in more personalized treatment plans, moving away from a one-size-fits-all approach. More effective treatment protocols and targeted resource allocation based on predictive factors can reduce healthcare costs. Monitoring IL-6 could become a standard part of ICU management, guiding interventions to modulate the immune response. Understanding the role of inflammatory markers like Il-6 may also have implications for other diseases characterized by systemic inflammation. Previous research has identified cytokine profiles, particularly elevated Il-6, as indicators of disease severity and poor prognosis in COVID-19 patients [13]. Our findings additionally indicate these markers directly with ICU mortality. Consequently, it highlights the potential of these markers as indicators for worsening conditions and prognosis in ICU settings.

Patients who survive long enough to develop certain conditions (coinfections) may differ fundamentally from those who die before these conditions occur. It could skew the results to make it appear as though these conditions are protective. Reverse Causality: The risk factor and mortality relationship is not unidirectional. For instance, the higher incidence of VAP in survivors infer that these patients had a longer period of mechanical ventilation, which increases the risk for VAP [21].

Our study has several limitations, including its retrospective and single-center design, which introduces inherent biases and limitations associated with the use of existing medical records. However, the large sample size and meticulous data collection strengthen the reliability of our conclusions.

## Conclusion

The study underscores the limited predictive value of traditional demographic and clinical factors for ICU mortality in COVID-19 patients, highlighting the critical role of dynamic immune response markers like Il-6 and WBC counts. These findings advocate for a more focused approach to early immune modulation in ICU management for COVID-19. It underscores the need for personalized treatment strategies and further research into the mechanisms of immune dysregulation in COVID-19.

## Supporting information

S1 File (DOCX)

S1 Data (XLSX)

## Abbreviations

BSI: Bloodstream Infection
VAP: Ventilator-Associated Pneumonia
MDR: Multi-Drug Resistant
MDRo: Multi-Drug Resistant Organisms
ICU: intensive care unit
DM: Diabetes Mellitus
AF: Atrial Fibrillation
HT: Hypertension
CHF: Chronic Heart Failure
APACHE II: Acute Physiology and Chronic Health Evaluation II
ICU: Intensive Care Unit
LOS: length of stay
MV: mechanical ventilation
APACHE II: Acute Physiology and Chronic Health Evaluation II
ARDS: Acute Respiratory Distress Syndrome
NMBAs: neuromuscular blocking agents
ECDC: European Centre for Disease and Control
