# Supplementary material for: Analysis of prognostic factors in critically ill patients with COVID-19
Source: PLoS One. 2024 Jun 27;19(6):e0302248. doi: 10.1371/journal.pone.0302248 (PMC11210843; doi:10.1371/journal.pone.0302248)
Supplement: Attachment — Submitted filename: Response to Reviewers.docx [file pone.0302248.s003.docx]

*Manuscript Title:* Analysis of prognostic factors in critically ill patients with COVID-19
*Authors:* Klaudia Bartoszewicz, Mateusz Bartoszewicz, Wojciech Gradkowski, Anna Stasiak-Barmuta, Sławomir Lech Czaban.
*Manuscript ID:* PONE-D-23-34297

Dear Dr Benjamin M. Liu and PLOS ONE Editorial Team

We appreciate the valuable feedback from the academic Editor and Reviewers on our manuscript, "Analysis of prognostic factors in critically ill patients with COVID-19." We have carefully considered each comment and made corresponding revisions to our manuscript. Below, we detail our responses to each of the points raised.

**Table 1.** Author response to Reviewers.

| **Reviewer comment** | **Author response** | **Page number** |
| --- | --- | --- |
| The manuscript failed to cite important review articles on risk factors like IL-6, cytokine storm, and ARDS. Information about the biomarkers and other indexes under investigation was not added. | Thank you for your feedback regarding including review articles on risk factors such as IL-6, cytokine storms, and ARDS, as well as the detailed information on the biomarkers and other indexes under investigation in our study. We have carefully revised the manuscript to address these concerns.  In the revised version, we have incorporated pertinent review articles and seminal studies that discuss the roles of IL-6, cytokine storms, and ARDS in COVID-19. These additions provide a more robust theoretical framework and align our findings with the current understanding of these critical aspects of the disease.  Furthermore, we have expanded the sections detailing the biomarkers and indexes we investigated. This includes a comprehensive explanation of their selection, their relevance in assessing the severity and progression of COVID-19, and their importance in the context of our study's objectives.  These revisions significantly strengthen the manuscript, offering a more complete and scientifically rigorous contribution to the field. Thank you again for your constructive comments, which have been instrumental in enhancing the quality of our work. | p.3, 7-10 |
| More information on methods of detecting SARS-CoV-2 and biomarkers. | We have updated our manuscript to include a detailed description of the methods for detecting SARS-CoV-2 and marking laboratory parameters. This new paragraph provides the diagnostic techniques for confirming COVID-19 infection in our patient cohort. Additionally, we have added information on the method of analysis of biomarkers in our study. | Methods Section |
| Question on whether statistical analysis is two-tailed or one-tailed. | Clarified the type of statistical analysis used. | p.5 |
| 1. Lack of Clarity in Variable Selection Protocol: Reference to Improve Analysis.  2. Concerns with APACHE II Score Analysis. Questionable Significance of APACHE II as a Predictor. Concerns with Mechanical Ventilation Duration Analysis. Ambiguity in MV Duration Analysis. Recommendation for Improved Data Analysis.  **3.**  Recommendation for improved conclusions section. | Variable Selection Protocol: Our revised manuscript defines the protocol for selecting variables for univariate and multivariate logistic regression analyses. This includes a rationale for each variable's inclusion, ensuring greater transparency and alignment with the best practices suggested in reference 9.  APACHE II Score Analysis: Upon reanalysis using logistic regression, the APACHE II scores did not emerge as significant predictors of in-ICU mortality. This finding is consistent with your observation regarding the similarity of APACHE II scores across different patient groups (Survivors, Non-Survivors, and overall). We have revised the manuscript to reflect this outcome, addressing the noted discrepancy.  Duration of Mechanical Ventilation (MV): Similarly, the reanalysis showed that the duration of MV is not significantly associated with in-ICU mortality. This aligns with your observation regarding the lack of diffrentance in MV duration across patient groups. The revised manuscript has been updated to represent these findings accurately.  These revisions have greatly enhanced our statistical analysis's clarity, accuracy, and validity. By addressing each of your concerns and incorporating a more appropriate statistical method, our study's conclusions are now more robust and reliable. We are grateful for your guidance in improving our manuscript and believe these changes have significantly strengthened our research. | p.4-10 |
| Incomplete sentence where reference 19 is cited. | We reviewed and corrected the incomplete sentence. | p.10 |

**Table 2.** Author response to Editor.

| **Editor Comment** | **Author response** | **Page number** |
| --- | --- | --- |
| Ensure the manuscript meets PLOS ONE's style requirements | Confirmed adherence to PLOS ONE's style requirements in the revised manuscript, including file naming conventions. | Entire Manuscript |
| Clarification on data availability upon request | Provided detailed explanation of ethical or legal restrictions for data sharing. | Methods Section |
| Requirement of an ORCID iD for the corresponding author | Confirmed that an ORCID iD for the corresponding author has been added and validated in Editorial Manager. | N/A |
| Relocation of ethics statement to the Methods section | Ensured that the ethics statement was appropriately placed only in the Methods section of the revised manuscript. | Methods Section |
| Use of PACE for figure file submission | Confirmed that all figure files have been uploaded and verified using the PACE digital diagnostic tool. | Figure 1 |

We hope that our responses and revisions adequately address the concerns raised. We believe these changes have significantly improved our manuscript, and we thank the Reviewers and academic Editor for their constructive feedback.

Sincerely,

PhD Sławomir Lech Czaban
Department of Anaesthesiology and Intensive Care, Medical University of Bialystok, Poland
e-mail: slawomir.czaban@umb.edu.pl

phone number: +48604486369
